# ERICA: sexual initiation and contraception in Brazilian adolescents

**DOI:** 10.1590/S01518-8787.2016050006686

**Published:** 2016-02-02

**Authors:** Ana Luiza Vilela Borges, Elizabeth Fujimori, Maria Cristina Caetano Kuschnir, Christiane Borges do Nascimento Chofakian, Ana Júlia Pantoja de Moraes, George Dantas Azevedo, Karine Ferreira dos Santos, Mauricio Teixeira Leite de Vasconcellos

**Affiliations:** I Departamento de Enfermagem em Saúde Coletiva. Escola de Enfermagem. Universidade de São Paulo. São Paulo, SP, Brasil; II Núcleo de Estudos da Saúde do Adolescente. Universidade do Estado do Rio de Janeiro. Rio de Janeiro, RJ, Brasil; III Programa de Pós-Graduação em Enfermagem. Escola de Enfermagem. Universidade de São Paulo. São Paulo, SP, Brasil; IV Faculdade de Medicina. Universidade Federal do Pará. Belém, PA, Brasil; V Departamento de Morfologia. Centro de Biociências. Universidade Federal do Rio Grande do Norte. Natal, RN, Brasil; VI Programa de Pós-Graduação em Saúde da Criança e do Adolescente. Faculdade de Medicina. Universidade Federal de Minas Gerais. Belo Horizonte, MG, Brasil; VII Escola Nacional de Ciências Estatísticas. Fundação Instituto Brasileiro de Geografia e Estatística. Rio de Janeiro, RJ, Brasil

**Keywords:** Adolescent, Sexual Behavior, Contraception, Sexual and Reproductive Health, Cross-Sectional Studies

## Abstract

**OBJECTIVE:**

To estimate the prevalence of sexual initiation and contraceptive use at the last sexual intercourse of Brazilian adolescents, according to sociodemographic features.

**METHODS:**

The data were obtained from the Study of Cardiovascular Risks in Adolescents (ERICA), a national school-based cross-sectional study. We included 74,589 adolescents from 32 geographic strata (27 capitals and five sets of municipalities with more than 100,000 inhabitants of each of the five macro-regions of the Country). Information on sexual initiation and contraceptive use at the last sexual intercourse (male condom and oral contraceptive pill) has been used. We have estimated prevalence and confidence intervals (95%CI) considering sample weights according to sex, age, type of school, residence status, macro-region and capitals.

**RESULTS:**

We observed that 28.1% (95%CI 27.0-29.2) of the adolescents had already initiated sexual life, with higher prevalence among those aged 17 years (56.4%, 95%CI 53.9-58.9), males (33.5%, 95%CI 31.8-35.2), studying at public schools (29.9%, 95%CI 28.5-31.4), and from the Northern region (33.9%, 95%CI 32.3-35.4), mainly from Macapa, Manaus, and Rio Branco. Among those who had started their sexual life, 82.3% (95%CI 81.1-83.4) reported the use of contraceptive methods at the last intercourse, and the prevalence of use was higher among adolescents aged 17 years (85.3%, 95%CI 82.7-87.6), females (85.2%, 95%CI 83.8-86.5) and those living in the Southern region (85.9%, 95%CI 82.9-88.5). Male condom was used by 68.8% (95%CI 66.9-70.7), with no difference by type of school or macro-regions; the contraceptive pill was used by 13.4% (CI95% 12.2-14.6), and more frequently used among women (24.7%, 95%CI 22.5-27,0) and 17-year-old adolescents (20.8%, 95%CI 18.2-23.6) from urban settings(13.7%, 95%CI 12.5-14.9) and from the Southern region (22.6%, 95%CI 19.0-26.8), and less often in the Northern region.

**CONCLUSIONS:**

ERICA’s data analysis on sexuality and contraception shows heterogeneities in the prevalence of sexual initiation and use of contraceptive methods among Brazilian adolescents, depending on their age, where they live, and the type of school they study at. Younger adolescents and those living in the Northern region seem to be more vulnerable to the consequences of unprotected sexual intercourses.

## INTRODUCTION

Recognizing adolescents as individuals with sexual and reproductive rights is essential for elaborating and introducing policies and programs that support individuals to have a safe transition from adolescence to adulthood. Among the experiences involving this transition, the experience of the first sexual intercourse is the focus of this study, especially because it creates specific needs of education for sexuality and contraception.

The first sexual intercourse is an event that occurs mainly during adolescence^8^ ^,^ ^17^ ^,^ ^20^. In Brazil, the National Adolescent School-based Health Survey (PeNSE) performed in 2009^11^ and 2012^16^ observed that, respectively, 20.5% and 28.7% of ninth-grade students (13-15 years old) had already initiated their sexual life. In addition, the adolescent’s gender, the region of residence and type of school have been described as determining factors for the sexual initiation^4^ ^,^ ^7^ ^,^ ^16^.

Health professionals are concerned about the onset of sexual life in adolescence because this event exposes adolescents to sexually transmitted diseases and HIV/AIDS, unplanned pregnancy and abortion. Thus, the use of contraceptives, particularly male condoms, is desirable and is one of the landmarks of a healthy sexuality during adolescence. Studies with adolescents reveal that contraception at the last intercourse varies between 75.0% and 86.0%^9^ ^,^ ^11^ ^,^ ^16^, of which male condoms and oral pills are the most used methods.

Despite the fact that numerous studies have already investigated sexual and reproductive health behaviors of adolescents, many are not nationally representative, i.e., restricted to adolescents from public schools or to younger age groups. Therefore, the objective of this study was to estimate the prevalence of sexual initiation and contraceptive use at last intercourse of Brazilian adolescents, according to sociodemographic characteristics.

## METHODS

The Study of Cardiovascular Risks in Adolescents (ERICA) is a national, school-based cross-sectional study, conducted to estimate the prevalence of cardiovascular risk factors in adolescents from schools in Brazilian cities with more than 100,000 inhabitants. The sample included both male and female students, aged 12 to 17 years, who attended the seventh, eighth and ninth grades of the elementary school and first, second and third grades of the high school in public and private institutions, urban and rural areas, in 2013 and 2014. The school grades chosen were intended to be *proxy* of the chronological age. Three classes of each school were selected, and all students invited to participate in the research.

The research population was stratified in 32 geographic strata, comprising the 27 Country’s State capitals and the Federal District and five groups of other municipalities with more than 100,000 inhabitants (large cities) of the five macro-regions of the Country. A sample of 1,251 schools was selected with probability proportional to size. So, ERICA’s sample has representativeness for the group of large cities in the national and regional level, for each capital and the Federal District. The sample calculation considered the 4.0% prevalence (with maximum error = 0.9%) of metabolic syndrome in adolescents and a 95% confidence level. In total, we selected 75,060 adolescents by sampling and cluster by school, grade and class. Among the schools selected, four refused the invitation, three in the state of Sao Paulo and one in the state of Amapa. Exclusion criteria were “having provisional or permanent disability that incapacitates the adolescent’s participation in the study (visual impairment, hearing impairment, intellectual disability, and physical disability) or an ongoing pregnancy”. Sample design details are found elsewhere^23^.

ERICA’s data were collected using standard techniques. Trained evaluators applied a 24-hour dietary recall and three questionnaires (one for adolescents, one for their parents and the other for schools’ principals). They also assessed anthropometric and blood pressure data, and conducted laboratory blood tests. The adolescent questionnaire was self-filled using an electronic data collector – personal digital assistant (PDA).

Besides specifically researching cardiovascular risk factors, ERICA addresses other aspects of adolescents’ lives, such as reproductive health, to contribute to future studies on the relationship between nutritional status, cardiovascular risks, menarche and use of oral hormonal contraceptives. The adolescent questionnaire included 11 blocks: socioeconomic characteristics, work, physical activity, eating behavior, smoking, alcohol consumption, oral health, common mental disorders, reproductive health, medical records of chronic diseases, and sleep^2^.

This study describes information only about the adolescents. It also estimates the prevalence and confidence intervals (95%CI) of the following variables: sexual initiation; use of contraceptives at last intercourse; use of male condom at last sexual intercourse; and use of oral contraceptive pill at last sexual intercourse. For these last two variables, only adolescents who reported having initiated sexual life were considered.

The prevalence and its respective confidence intervals (95%CI) were estimated by gender (male; female), age (12, 13, 14, 15, 16, and 17 years), school type (public; private), residence status (urban; rural), and macro-region of residence (North, Northeast, Midwest, Southeast and South), State capitals and Federal District.

All analyses were performed considering sample weights, calculated by the product of the inverse of inclusion probabilities in each sample stratum and calibrated by the estimated population of adolescents enrolled in schools located in the geographic strata by gender and age. Data were analyzed in Stata Statistical Software 13.0.

The study was approved by Research Ethics Committees of the 27 States and Federal District. All adolescents signed an informed assent form. Their parents also signed and informed consent form where it was required (States of Bahia, Goias, Mato Grosso do Sul, Minas Gerais, and Roraima).

## RESULTS

**Figure 1:**
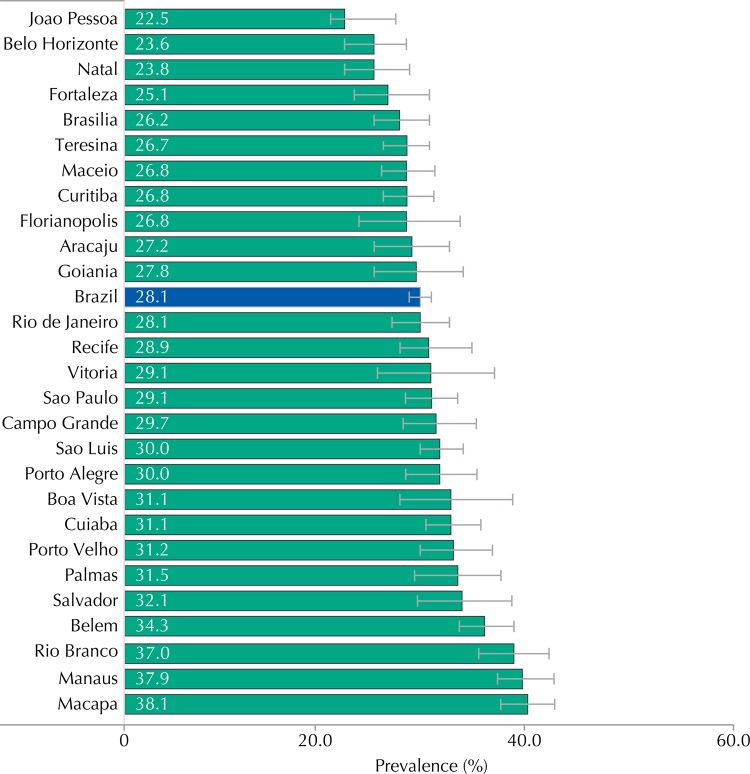
Prevalence of sexual initiation among adolescents, according to capitals. ERICA, Brazil, 2013-2014.

We considered data of 74,589 adolescents. The sexual initiation was reported by 28.1% (95%CI 27.0-29.2), with increasing prevalence along the ages. The prevalence of sexual initiation reached 56.4% among those aged 17 years (95%CI 53.9-58.9), and was significantly higher in male adolescents (33.5%; 95%CI 31.8-35.2), from public schools (29.9%; 95%CI 28.5-31.4) and residents in the Northern region (33.9%; 95%CI 32.3-35.4) (Table). In relation to the distribution by capitals, it ranged from 22.5% (95%CI 19.7-25.6) in Joao Pessoa to 38.1% (95%CI 35.5-40.9) in Macapa (Figure 1).

**Table:** Prevalence (%) of sexual initiation and use of contraceptives at the last sexual intercourse, according to sociodemographic variables. ERICA, Brazil, 2013-2014. (N = 74,589)

Sociodemographic variable	Initiated the sexual life		Used contraceptive method at the last sexual intercourse		Used male condom at the last sexual intercourse*		Used oral pill at last sexual intercourse*	
			
	%	95%CI	%	95%CI	%	95%CI	%	95%CI
Sex								
Male	33.5	31.8-35.2	80.3	78.7-81.9	70.9	68.7-73.1	5.8	4.9-6.9
Female	22.6	21.7-23.6	85.2	83.8-86.5	65.7	62.9-68.3	24.7	22.5-27.0
Age (years)								
12	5.9	4.9-7.0	67.1	60.0-73.4	46.9	40.5-53.4	2.3	0.8-5.9
13	12.1	10.6-13.8	74.9	70.8-78.6	60.5	55.2-65.7	3.0	1.9-4.8
14	21.4	20.0-22.9	81.4	78.2-84.3	72.6	68.8-76.1	6.8	5.4-8.7
15	35.9	33.4-38.5	81.6	79.1-83.8	70.4	67.0-73.7	11.3	9.5-13.4
16	44.2	40.1-47.5	84.4	82.2-86.3	70.6	68.0-73.1	15.3	13.3-17.5
17	56.4	53.9-58.9	85.3	82.7-87.6	69.2	64.6-73.4	20.8	18.2-23.6
Type of school								
Public	29.9	28.5-31.4	82.2	80.9-83.4	70.0	67.0-70.9	12.9	11.7-14.3
Private	19.4	15.1-24.5	82.9	79.3-85.9	67.7	61.2-73.6	16.8	13.9-20.0
Place of residence								
Urban	28.4	27.2-29.6	82.3	81.1-83.4	68.7	66.7-70.6	13.7	12.5-14.9
Rural	19.8	11.3-32.4	81.6	74.6-87.1	74.7	70.4-78.6	3.8	1.5-9.2
Macro-region								
North	33.9	32.3-35.4	79.8	78.4-81.1	69.4	67.6-71.2	6.6	5.8-7.6
Northeast	26.9	24.4-29.5	78.0	75.6-80.3	64.9	61.5-68.1	9.0	7.6-10.7
Midwest	29.3	26.8-32.0	83.1	80.9-85.0	71.2	67.8-74.4	12.9	10.9-15.1
Southeast	27.7	26.1-29.4	83.5	81.5-85.4	69.8	66.4-73.0	14.6	12.6-16.7
South	26.9	24.1-29.9	85.9	82.9-88.5	69.5	65.3-73.4	22.6	19.0-26.8
Brazil	28.1	27.0-29.2	82.3	81.1-83.4	68.8	66.9-70.7	13.4	12.2-14.6

* Considered only adolescents who had already started their sexual life.

**Figure 2:**
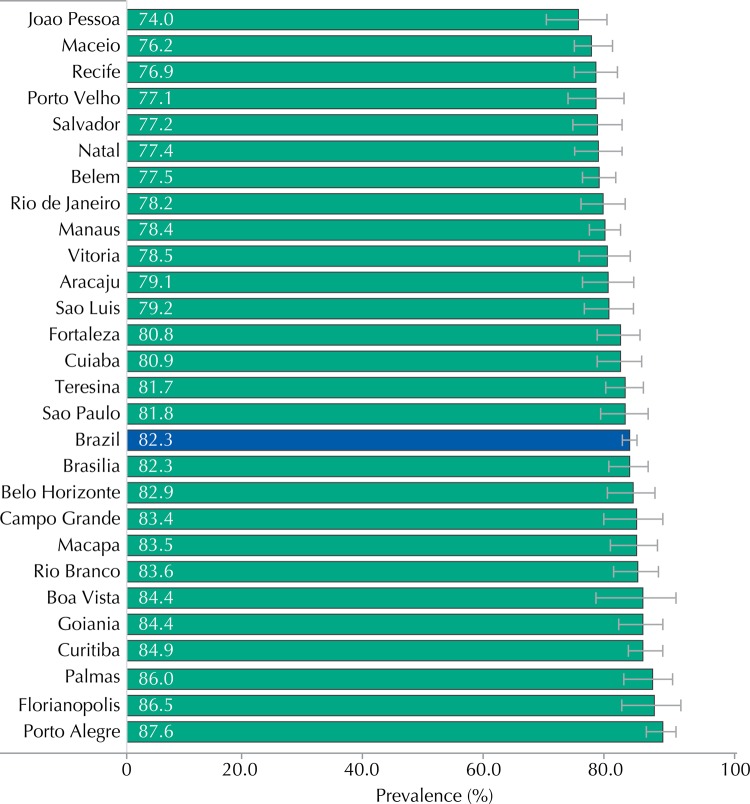
Prevalence of contraceptive use at the last sexual intercourse, according to capitals. ERICA, Brazil, 2013-2014.

Considering the adolescents who had already had their first intercourse (n = 22,241), 82.3% (95%CI 81.1-83.4) used contraceptives at the last sexual intercourse. The use of contraceptives at the last sexual intercourse was significantly higher among female adolescents (85.2%, 95%CI 83.8-86.5), aged 16 and 17 years (84.4% and 85.3%, respectively) and residents in the Southern region (85.9%, 95%CI 82.9-88.5) (Table). Porto Alegre was the capital where most adolescents reported the use of contraceptives at the last intercourse (87.6%, 95%CI 85.0-89.8), while Joao Pessoa (74.0%, 95%CI 68.7-78.7) was the capital where this event was less reported (Figure 2).

**Figure 3:**
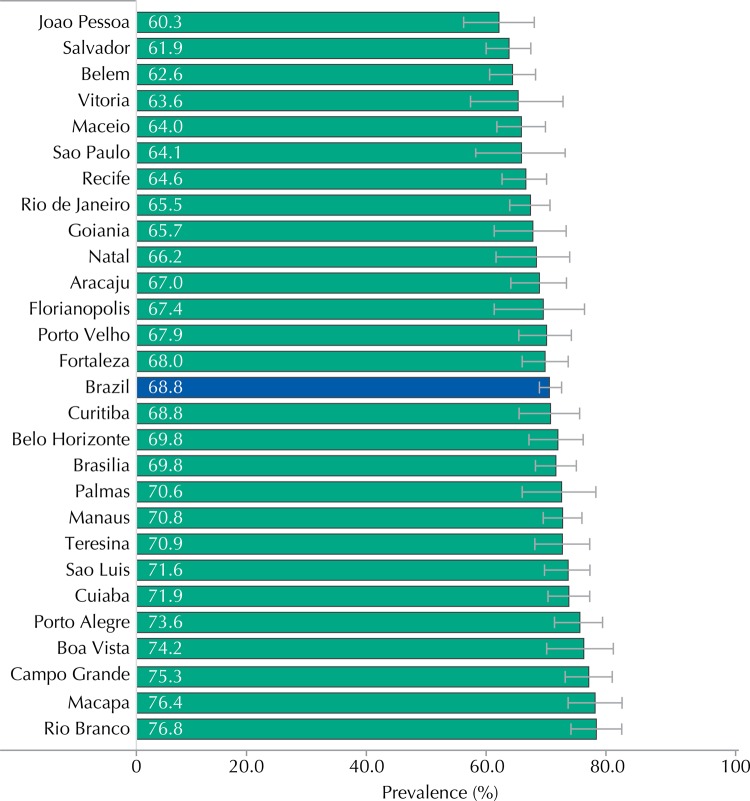
Prevalence of male condom use at the last sexual intercourse, according to capitals. ERICA, Brazil, 2013-2014.

Male condom was used by 68.8% (95%CI 66.9-70.7) of the adolescents at the last sexual intercourse (Table). No statistically significant difference was observed in the prevalence of condom use among adolescents from public and private schools and in the several macro-regions of the Country. However, female adolescents (65.7%, 95%CI 62.9-68.3), younger individuals (46.9% among adolescents aged 12 years; 95%CI 40.5-53.4) and residents of urban areas (68.7, 95%CI 66.7-70.6) were those for whom we identified a lower prevalence of male condom use at last sexual intercourse. The use of condom ranged from 60.3% (95%CI 54.4-65.9) in Joao Pessoa to 76.8% (95%CI 72.3-80.1) in Rio Branco (Figure 3).

**Figure 4:**
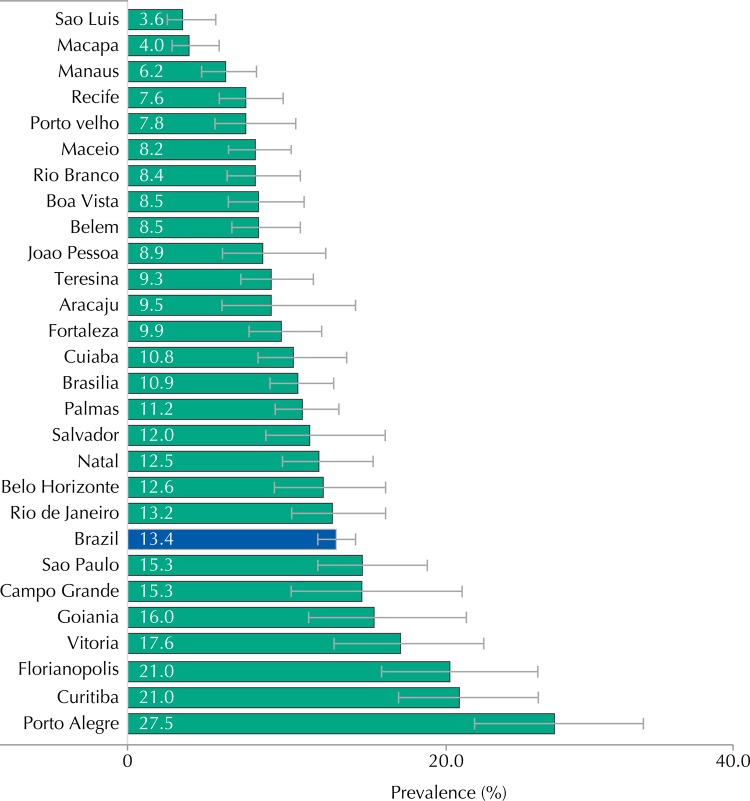
Prevalence of oral pill use at the last sexual intercourse, according to capitals. ERICA, Brazil, 2013-2014.

Use of oral pills at the last sexual intercourse was much less frequent (13.4%, 95%CI 12.2-14.2), even though the prevalence was very heterogeneous when we compared sociodemographic characteristics. Its reported use was statistically higher among female adolescents (24.7%, 95%CI 22.5-27.0), older ones (20.8% in those aged 17 years, 95%CI 18.2-23.6), residents in urban areas (13.7%, 95%CI 12.5-14.9) and in the Southern region (22.6%, 95%CI 19.0-26.8) (Table). Figure 4 shows a great heterogeneity for the prevalence of oral pills at the last sexual intercourse among the Capitals, especially in Sao Luis, with 3.6% (95%CI 2.4-5.4), and Porto Alegre, with 27.5% (95%CI 22.4-33.2).

## DISCUSSION

ERICA’s data analysis, which included a national sample of Brazilian students aged 12 to 17 years, living in a country of continental dimensions and multicultural and social varied background, indicates that these events do not occur homogeneously among adolescents, considering gender, age, type of school where they study, situation, macro-region and capital of residence. Data showed that behaviors related to sexual initiation and the use of contraceptives at last sexual intercourse are influenced by sociodemographic characteristics.

More than one-fifth of adolescents aged 12 to 17 years have already had their first sexual intercourse in Brazil. The comparison with other national studies is complex, because PeNSE^16^ has assessed only ninth-grade students, that is, younger adolescents, and the Demographic and Health Survey 2006^13^ interviewed only women aged from 15 years on. However, we can conclude that the prevalence estimated herein is consistent with these studies. The prevalence in Brazil is higher than that of high-income countries, such as Spain and United States; however, it is lower when compared to some African countries, whose prevalence reaches 39.0%^5^ ^,^ ^9^ ^,^ ^10^.

A trend of increasing prevalence of sexual initiation as the age increases was also confirmed, as observed in other studies^4^ ^,^ ^7^ ^,^ ^16^. As expected, at 17 years of age, more than half of adolescents had already started their sexual life, since it is an event that tends to occur from the age of 15 on^20^ ^,^ ^21^. We need to consider, however, that the much lower prevalence of sexual initiation observed among adolescents aged 12 to 14 years does not minimize its importance. On the contrary, it reinforces the need of sexual education in the early years of adolescence. So sexual education should be included in policies, programs and practices of adolescent health care to protect their sexual and reproductive rights and to assist a healthy onset of a sexual life, with responsibility and free of any coercion.

Both the residence status (urban or rural) and the macro-region of residence showed differences in the prevalence of sexual initiation − adolescents living in the Northern region are the ones who reported more frequently having initiated the sexual life. In fact, according to a national survey^14^, the Northern region had the highest percentage of adolescents who initiated sexual life before the age of 15. PeNSE 2012 results also confirm the heterogeneity in the prevalence of sexual initiation according to the macro-region of residence^16^. How cultural and social characteristics of the different Brazilian regions influence on initiating sexual life and their consequences in the health of this group still need to be clarified.

The prevalence of adolescents’ sexual initiation was statistically different for boys and girls. This was expected, since it is an event in which gender relations have an indubitable role. Adolescents begin to have sex motivated by current standards of sexual behaviors that differentiate male and female roles in relation to the most appropriate time for sexual initiation^3^ ^,^ ^12^ ^,^ ^20^. The prevalence of sexual initiation was higher among students from public schools, which indirectly seems to ratify the differences in sexual behavior between socioeconomic strata reported in other studies^7^ ^,^ ^22^.

Regarding contraceptive use at last sexual intercourse, most adolescents reported having used a method, which is also in line with other studies^1^ ^,^ ^16^. Measuring contraceptive use at the last sexual intercourse is an effective strategy to comprehend contraceptive behavior during adolescence, given the sporadic nature of sexual relations, the dynamics of the affective relationships that are established at this stage and the consequent switch of methods. The prevalence of contraceptive use at last sexual intercourse was relatively high, with statistically significant differences between gender, age and macro-region, indicating that there are sexually active groups more vulnerable to the consequences of unprotected sex, such as the younger adolescents and those who live in the Northern region.

In the Brazilian context, there is a consensus adolescents’ contraceptive use is basically limited to male condom and oral pill^3^ ^,^ ^6^ ^,^ ^24^. In this sense, it is very positive that most adolescents have used male condom at the last sexual intercourse, as shown by other national surveys;^16^ mainly because it is also a method of preventing HIV/AIDS and other sexually transmitted diseases. However, this prevalence still needs to be expanded, as it is much lower than the 80.0% observed in most European countries^20^.

In addition, little is known if adolescents correctly and constantly use condoms. In fact, in the adolescent group, there is a tendency of stop using condoms when the relationship becomes stable. It seems that young people tend to be less vigilant when they are involved in long-lasting relationships^1^. Consequently, according to Pirotta and Schor^18^ (2004), male condom is used mainly in casual relationships and in the beginning of relationships with a new partner, as adolescents can neglect the male condom in long-lasting and stable relationships, and tend to replace it by the pill. If our findings are analyzed in the perspective of preventing a pregnancy rather than just preventing HIV and AIDS, the high prevalence of condom use may be translated into potential risks of failures and discontinuities, since its efficacy depends on the individual ability to use it correctly and consistently. Unfortunately, this ability is not always observed, especially regarding the younger and inexperienced adolescents^19^.

Due to a major concern about the prevention of HIV/AIDS and other sexually transmitted diseases, the most recent nationwide studies focusing on contraceptive use among adolescents do not describe the use of oral pills, as we have done in this study^11^ ^,^ ^16^. Surely, this is the potentiality of our findings when comparing them to other nationwide studies conducted with adolescents. Except for the type of school, all the other sociodemographic characteristics were associated with the use of oral pill in our study.

However, our most interesting finding is the difference in the prevalence of oral pill use in the Northern and Northeastern regions in relation to other macro-regions. This can be explained by the fact that one of the key elements in the use of contraceptives is the access to health services and contraceptive supplies. Male condoms are freely distributed in most basic health facilities in Brazil, and do not require a medical prescription or any other prerequisite, even for adolescents. In its turn, the process of obtaining the pill can be more complex, because, as it is a hormonal method, its distribution in the Country is still permeated by the formal requirement of a prescription^15^. In the specific case of adolescents, this requirement can become a barrier to obtain the pill, unless health services are sufficiently organized to consider the need of contraceptive methods of this group. Our hypothesis is that, in areas where the barriers to obtain the oral pill are not so evident, the use of pill is higher. However, we do not refute that the acquisition of the pill may have occurred in pharmacies and drugstores, in which the requirement for prescription is flexible and the prices are relatively low. In this case, it is clear that adolescents could somehow afford the costs of purchasing the pill, which may not be possible for everyone, depending on the socioeconomic strata to which they belong. In any case, our findings do not allow us to understand how the process of obtaining the contraceptive was or whether there was any kind of contraceptive counseling to promote the correct and continuous use.

The results reveal singularities in the sexual behavior and contraceptive use of Brazilian adolescent students. The Northern region, e.g., had the higher prevalence of sexual initiation, while the prevalence of contraceptive use, in particular, the oral pill, is one of the lowest among the macro-regions. The analyses by capital also show this disquieting contradiction: Northern capitals are among those whose adolescents most often reported having initiated the sexual life, although reports of contraceptive use, especially oral pill, do not follow the same trend. The sexual and reproductive health policies must consider such differences and regional specificities in the adolescent health care. National programs such as the Health at School (PSE), which requires the integration of schools and primary health services, need to be fully established to contemplate all the students and also their health needs, as the school environment can be a place of relevant changes in the life and health of adolescents. Ultimately, this could help to ensure that the first sexual experiences of young people are healthy, i.e., desired, protected, and enjoyable.

The limitations of this study rely on the fact that some singularities of the adolescent group in relation to sexual behavior and contraception involve individual elements that have not been explored, such as the age at the first sexual intercourse, sexual experience, affective involvement and relational stability^1^ ^,^ ^18^. In addition, although ERICA is a nationwide study, only adolescent students of large cities were considered and, therefore, it does not represent those living in towns with populations less than 100,000 inhabitants. Also, ERICA did not include the adolescents who are out of schools. This fact may have caused some underestimation of the estimates, because these adolescents can be the older ones, worker or even women who have already experienced motherhood, i.e., with distinct sexual behavior.

In addition, some sexual behaviors may have been under- or overreported to meet socially acceptable patterns. The use of self-filling forms with PDA may have helped to minimize this bias. Anyway, it is a limitation inherent to studies covering personal and intimate questions among adolescents. Although the study has such limitations, we confirmed it contributes to the topic of sexuality and contraception in adolescence.

In conclusion, ERICA’s data on sexuality and contraception show that there are heterogeneities in the prevalence of sexual initiation and use of contraceptives, such as the male condom and oral contraceptive pill, among Brazilian adolescents, depending on their age, where they live and the type of school they attend. Younger adolescents and those living in the Northern region seem to be the most vulnerable to the consequences of unprotected sexual intercourses.
