# Supplementary material for: ERICA: sexual initiation and contraception in Brazilian adolescents
Source: Rev Saude Publica. 2016 Feb 2;50(Suppl 1):15s. doi: 10.1590/S01518-8787.2016050006686 (PMC4767039; doi:10.1590/S01518-8787.2016050006686)
Supplement: Translation

# Translation: ERICA: início da vida sexual e contracepção em adolescentes brasileiros

**Authors:** Ana Luiza Vilela Borges, Elizabeth Fujimori, Maria Cristina Caetano Kuschnir, Christiane Borges do Nascimento Chofakian, Ana Júlia Pantoja de Moraes, George Dantas Azevedo, Karine Ferreira dos Santos, Mauricio Teixeira Leite de Vasconcellos

**Affiliations:** I Departamento de Enfermagem em Saúde Coletiva. Escola de Enfermagem. Universidade de São Paulo. São Paulo, SP, Brasil; II Núcleo de Estudos da Saúde do Adolescente. Universidade do Estado do Rio de Janeiro. Rio de Janeiro, RJ, Brasil; III Programa de Pós-Graduação em Enfermagem. Escola de Enfermagem. Universidade de São Paulo. São Paulo, SP, Brasil; IV Faculdade de Medicina. Universidade Federal do Pará. Belém, PA, Brasil; V Departamento de Morfologia. Centro de Biociências. Universidade Federal do Rio Grande do Norte. Natal, RN, Brasil; VI Programa de Pós-Graduação em Saúde da Criança e do Adolescente. Faculdade de Medicina. Universidade Federal de Minas Gerais. Belo Horizonte, MG, Brasil; VII Escola Nacional de Ciências Estatísticas. Fundação Instituto Brasileiro de Geografia e Estatística. Rio de Janeiro, RJ, Brasil

**Keywords:** Adolescente, Comportamento Sexual, Anticoncepção, Saúde Sexual e Reprodutiva, Estudos Transversais

## Abstract

**OBJETIVO:**

Estimar prevalências de iniciação sexual e uso de métodos contraceptivos na última relação sexual de adolescentes brasileiros, segundo características sociodemográficas.

**MÉTODOS:**

Os dados foram obtidos do Estudo de Riscos Cardiovasculares em Adolescentes (ERICA), estudo transversal nacional de base escolar. Foram incluídos 74.589 adolescentes provenientes de 32 estratos geográficos (27 capitais e cinco conjuntos de municípios com mais de 100 mil habitantes de cada uma das cinco macrorregiões do País). Utilizaram-se informações sobre iniciação sexual e uso de métodos contraceptivos na última relação sexual (preservativo masculino e pílula anticonceptiva oral). Estimaram-se prevalências e intervalos de confiança (IC95%) das variáveis de interesse considerando pesos amostrais e segundo sexo, idade, tipo de escola, situação de residência, macrorregião e capitais.

**RESULTADOS:**

Observou-se que 28,1% (IC95% 27,0-29,2) dos adolescentes tinham iniciado a vida sexual, com maior prevalência naqueles com 17 anos (56,4%, IC95% 53,9-58,9), no sexo masculino (33,5%, IC95% 31,8-35,2), em escolas públicas (29,9%, IC95% 28,5-31,4) e na região Norte (33,9%, IC95% 32,3-35,4), destacando-se em Macapá, Manaus e Rio Branco. Entre adolescentes que tinham iniciado a vida sexual, 82,3% (IC95% 81,1-83,4) referiram uso de métodos contraceptivos na última relação sexual, sendo a prevalência de uso maior entre adolescentes com 17 anos de idade (85,3%, IC95% 82,7-87,6), mulheres (85,2%, IC95%:83,8-86,5) e residentes na região Sul (85,9%, IC95% 82,9-88,5). O preservativo masculino foi usado por 68,8% (IC95% 66,9-70,7), sem diferença por tipo de escola ou macrorregiões. Pílula anticoncepcional foi utilizada por 13,4% (IC95% 12,2-14,6), sendo mais frequente entre mulheres (24,7%, IC95% 22,5-27,0), adolescentes de 17 anos (20,8%, IC95% 18,2-23,6), da área urbana (13,7%, IC95% 12,5-14,9) e da região Sul (22,6%, IC95% 19,0-26,8), e menos frequente na região Norte.

**CONCLUSÕES:**

A análise dos dados sobre sexualidade e contracepção do ERICA mostra que há heterogeneidades nas prevalências de iniciação sexual e uso de métodos contraceptivos entre os adolescentes brasileiros, a depender de sua idade, de onde vivem e do tipo de escola que frequentam. Adolescentes mais novos e residentes na região Norte parecem ser os mais vulneráveis às consequências das práticas sexuais não protegidas.

## INTRODUÇÃO

O reconhecimento dos adolescentes como sujeitos de direitos sexuais e reprodutivos é essencial para a construção e efetivação de políticas e programas que subsidiem os indivíduos a terem uma passagem segura pela adolescência rumo à vida adulta. Dentre as experiências que constituem essa transição, a vivência das primeiras práticas sexuais é o foco deste estudo, justamente porque gera nos adolescentes necessidades específicas de educação para a sexualidade e contracepção.

O início da vida sexual é um evento que tende a ocorrer majoritariamente durante a adolescência^8^ ^,^ ^17^ ^,^ ^20^. No Brasil, a Pesquisa Nacional de Saúde do Adolescente (PeNSE) de 2009^11^ e a de 2012^16^ observaram, respectivamente, que 20,5% e 28,7% dos estudantes do nono ano (13-15 anos) já tinham iniciado a vida sexual. Adicionalmente, o sexo do adolescente, a região de residência e o tipo de escola que frequenta têm sido descritos como determinantes do início da vida sexual^4^ ^,^ ^7^ ^,^ ^16^.

Profissionais de saúde preocupam-se com o início da vida sexual na adolescência porque esse evento insere os adolescentes em contextos de vulnerabilidades às doenças sexualmente transmissíveis e HIV/Aids, gestação não planejada e aborto. Assim, o uso de métodos contraceptivos, principalmente de preservativo masculino, é desejável e constitui-se em um dos marcos da vivência saudável da sexualidade na adolescência. Estudos realizados com adolescentes revelam que o uso de métodos contraceptivos na última relação sexual varia entre 75,0% e 86,0%^9^ ^,^ ^11^ ^,^ ^16^, sendo o preservativo masculino e a pílula oral os mais utilizados.

Apesar da existência de estudos que tenham investigado aspectos da saúde sexual e reprodutiva dos adolescentes, muitos carecem de representatividade nacional, restringem-se a adolescentes estudantes de escolas públicas ou incorporam apenas faixas etárias mais jovens. O objetivo deste estudo foi estimar as prevalências de iniciação sexual e uso de métodos contraceptivos na última relação sexual de adolescentes brasileiros, segundo características sociodemográficas.

## MÉTODOS

O Estudo de Riscos Cardiovasculares em Adolescentes (ERICA) é um estudo transversal, nacional, de base escolar, conduzido com o objetivo de estimar a prevalência de fatores de risco cardiovascular em adolescentes que frequentam escolas em cidades brasileiras com mais de 100 mil habitantes. A amostra incluiu estudantes de ambos os sexos, na faixa etária de 12 a 17 anos, que frequentavam o sétimo, oitavo e nono anos do ensino fundamental e primeiro, segundo e terceiro anos do ensino médio de escolas públicas e privadas, de áreas urbanas e rurais, em 2013 e 2014. Os anos escolares escolhidos tinham como finalidade serem *proxy* da idade cronológica. Foram selecionadas três turmas por escola e todos os alunos dessas turmas foram convidados a participar da pesquisa.

A população de pesquisa foi estratificada em 32 estratos geográficos, constituídos pelas 27 capitais dos estados e Distrito Federal e cinco conjuntos dos demais municípios com mais de 100 mil habitantes (municípios de grande porte) das cinco macrorregiões do País e uma amostra de 1.251 escolas foi selecionada com probabilidade proporcional ao tamanho. Assim, a amostra do ERICA tem representatividade para o conjunto de municípios de grande porte em nível nacional e regional, para cada capital e para o Distrito Federal (DF). O cálculo amostral considerou prevalência de síndrome metabólica em adolescentes de 4,0% com erro máximo de 0,9% e nível de confiança de 95%. No total, foram selecionados 75.060 adolescentes por meio de amostragem por conglomerado por escola, turno, ano e turma. Dentre as escolas selecionadas, quatro recusaram o convite, sendo três em São Paulo e uma no Amapá. Os critérios de exclusão foram apresentar qualquer deficiência provisória ou definitiva que incapacitasse a participação do adolescente no estudo (deficiência visual, deficiência auditiva, retardo mental aparente e deficiência física) ou gravidez em curso. Detalhes do desenho da amostra encontram-se em artigo publicado^23^.

Os dados do ERICA foram coletados utilizando-se técnicas padronizadas. Avaliadores treinados aplicaram recordatório alimentar de 24 horas e mais três questionários (um para adolescentes, um para responsáveis e outro para escolas), aferiram dados antropométricos e de pressão arterial e realizaram exames laboratoriais de sangue. O questionário para adolescente foi autopreenchido usando-se um coletor eletrônico de dados, Personnal Digital Assistant (PDA).

Além de o ERICA incluir especificamente os fatores de risco cardiovascular, aborda outros aspectos da vida dos adolescentes, como a saúde reprodutiva, com vistas a subsidiar estudos futuros sobre a relação entre estado nutricional, riscos cardiovasculares, menarca e uso de contraceptivos hormonais orais. O questionário dos adolescentes incluiu 11 blocos: características socioeconômicas, trabalho, atividade física, comportamento alimentar, fumo, consumo de álcool, saúde oral, transtornos mentais comuns, saúde reprodutiva, histórico médico de doenças crônicas e sono^2^.

O presente estudo descreve apenas informações dos adolescentes. Estimou-se prevalências e respectivos intervalos de confiança (IC95%) das seguintes variáveis: início da vida sexual; uso de métodos contraceptivos na última relação sexual; uso de preservativo masculino na última relação sexual; e uso da pílula anticonceptiva oral na última relação sexual. Para estas duas últimas variáveis, considerou-se apenas adolescentes que relataram ter iniciado a vida sexual.

As prevalências e seus respectivos intervalos de confiança (IC95%) foram estimados por sexo (masculino; feminino), idade (12, 13, 14, 15, 16 e 17 anos), tipo de escola (pública; privada), situação de residência (urbana; rural), macrorregião de residência (Norte, Nordeste, Centro-Oeste, Sudeste e Sul), capitais dos estados e Distrito Federal.

Todas as análises foram realizadas levando-se em conta os pesos amostrais, calculados pelo produto dos inversos das probabilidades de inclusão em cada estrato da amostra e calibrados considerando uma estimativa da população de adolescentes matriculados em escolas localizadas nos estratos geográficos considerados por sexo e idade. Os dados foram analisados no programa Stata versão 13.0.

O estudo foi aprovado por Comitês de Ética em Pesquisa dos 27 estados e DF. Todos os adolescentes assinaram o termo de assentimento, ou termo de consentimento livre e esclarecido também assinado pelos pais ou responsáveis, nos estados onde isso foi exigido (Bahia, Goiás, Mato Grosso do Sul, Minas Gerais e Roraima).

## RESULTADOS

Ao todo, foram considerados dados de 74.589 adolescentes. O início da vida sexual foi relatado por 28,1% (IC95% 27,0-29,2) dos adolescentes, com prevalências crescentes ao longo das idades consideradas e chegando a 56,4% entre aqueles com 17 anos (IC95% 53,9-58,9). As prevalências de início da vida sexual foram significativamente maiores nos adolescentes do sexo masculino (33,5%; IC95% 31,8-35,2), de escolas públicas (29,9%; IC95% 28,5-31,4) e residentes na região Norte (33,9%; IC95% 32,3-35,4) (Tabela). Em relação à distribuição por capitais, as prevalências de início da vida sexual variaram de 22,5% (IC95% 19,7-25,6) em João Pessoa a 38,1% (IC95% 35,5-40,9) em Macapá (Figura 1).

**Tabela:** Prevalência de início da vida sexual e uso de métodos contraceptivos na última relação sexual, segundo variáveis sociodemográficas. ERICA, Brasil, 2013-2014. (N = 74.589)

Variável sociodemográfica	Iniciou a vida sexual		Usou método contraceptivo na última relação sexual		Usou preservativo masculino na última relação sexual*		Usou pílula anticoncepcional na última relação sexual*	
			
	%	IC95%	%	IC95%	%	IC95%	%	IC95%
Sexo								
Masculino	33,5	31,8-35,2	80,3	78,7-81,9	70,9	68,7-73,1	5,8	4,9-6,9
Feminino	22,6	21,7-23,6	85,2	83,8-86,5	65,7	62,9-68,3	24,7	22,5-27,0
Idade (anos)								
12	5,9	4,9-7,0	67,1	60,0-73,4	46,9	40,5-53,4	2,3	0,8-5,9
13	12,1	10,6-13,8	74,9	70,8-78,6	60,5	55,2-65,7	3,0	1,9-4,8
14	21,4	20,0-22,9	81,4	78,2-84,3	72,6	68,8-76,1	6,8	5,4-8,7
15	35,9	33,4-38,5	81,6	79,1-83,8	70,4	67,0-73,7	11,3	9,5-13,4
16	44,2	40,1-47,5	84,4	82,2-86,3	70,6	68,0-73,1	15,3	13,3-17,5
17	56,4	53,9-58,9	85,3	82,7-87,6	69,2	64,6-73,4	20,8	18,2-23,6
Tipo de escola								
Pública	29,9	28,5-31,4	82,2	80,9-83,4	70,0	67,0-70,9	12,9	11,7-14,3
Privada	19,4	15,1-24,5	82,9	79,3-85,9	67,7	61,2-73,6	16,8	13,9-20,0
Local de residência								
Urbano	28,4	27,2-29,6	82,3	81,1-83,4	68,7	66,7-70,6	13,7	12,5-14,9
Rural	19,8	11,3-32,4	81,6	74,6-87,1	74,7	70,4-78,6	3,8	1,5-9,2
Macrorregião								
Norte	33,9	32,3-35,4	79,8	78,4-81,1	69,4	67,6-71,2	6,6	5,8-7,6
Nordeste	26,9	24,4-29,5	78,0	75,6-80,3	64,9	61,5-68,1	9,0	7,6-10,7
Centro-Oeste	29,3	26,8-32,0	83,1	80,9-85,0	71,2	67,8-74,4	12,9	10,9-15,1
Sudeste	27,7	26,1-29,4	83,5	81,5-85,4	69,8	66,4-73,0	14,6	12,6-16,7
Sul	26,9	24,1-29,9	85,9	82,9-88,5	69,5	65,3-73,4	22,6	19,0-26,8
Brasil	28,1	27,0-29,2	82,3	81,1-83,4	68,8	66,9-70,7	13,4	12,2-14,6

* Considerados apenas adolescentes que já iniciaram a vida sexual.

**Figura 1:** Prevalência de início da vida sexual entre adolescentes, segundo as capitais. ERICA, Brasil, 2013-2014.

Dos adolescentes que já haviam iniciado a vida sexual (n = 22.241), 82,3% (IC95% 81,1-83,4) usaram métodos contraceptivos na última relação sexual. O uso de métodos contraceptivos na última relação sexual foi significativamente maior por adolescentes do sexo feminino (85,2%, IC95% 83,8-86,5), de 16 e 17 anos de idade (84,4% e 85,3%, respectivamente) e residentes na região Sul (85,9%, IC95% 82,9-88,5) (Tabela). Porto Alegre foi a capital onde os adolescentes mais referiram ter usado método contraceptivo na última relação sexual (87,6%, IC95% 85,0-89,8), ao passo que João Pessoa (74,0%, IC95% 68,7-78,7) foi a capital onde esse evento foi menos relatado (Figura 2).

**Figura 2:** Prevalência de uso de método contraceptivo na última relação sexual, segundo as capitais. ERICA, Brasil, 2013-2014.

Preservativo masculino foi usado por 68,8% (IC95% 66,9-70,7) dos adolescentes na última relação sexual (Tabela). Não foi observada diferença estatisticamente significativa na prevalência de uso do preservativo entre adolescentes de escolas públicas e privadas e nas diversas macrorregiões do País. No entanto, adolescentes do sexo feminino (65,7%, IC95% 62,9-68,3), mais jovens (46,9% entre adolescentes com 12 anos de idade; IC95% 40,5-53,4) e residentes em área urbana (68,7, IC95% 66,7-70,6) foram aqueles em que se identificou menor prevalência de uso do preservativo masculino na última relação sexual. As prevalências de uso desse método segundo a capital de residência variaram de 60,3% (IC95% 54,4-65,9) em João Pessoa a 76,8% (IC95% 72,3-80,1) em Rio Branco (Figura 3).

**Figura 3:** Prevalência de uso de preservativo masculino na última relação sexual, segundo as capitais. ERICA, Brasil, 2013-2014.

O uso da pílula anticoncepcional oral na última relação sexual foi bem menos frequente, referida por 13,4% dos adolescentes brasileiros (IC95% 12,2-14,6), embora as prevalências tenham sido mais discrepantes nos diversos perfis sociodemográficos. Seu uso referido foi estatisticamente maior entre as mulheres (24,7%, IC95% 22,5-27,0), adolescentes mais velhos (20,8% naqueles com 17 anos, IC95% 18,2-23,6), residentes em área urbana (13,7%, IC95% 12,5-14,9) e na região Sul (22,6%, IC95% 19,0-26,8) (Tabela). Na Figura 4, observa-se grande heterogeneidade nas prevalências de uso da pílula oral na última relação sexual entre as capitais, com destaque para São Luís, com 3,6% (IC95% 2,4-5,4), e Porto Alegre, com 27,5% (IC95% 22,4-33,2).

**Figura 4:** Prevalência de uso de pílula anticoncepcional oral na última relação sexual, segundo as capitais. ERICA, Brasil, 2013-2014.

## DISCUSSÃO

A análise dos dados do ERICA, que contemplou amostra nacional de estudantes brasileiros de 12 a 17 anos de idade, residentes em um país de dimensões continentais e de formação social e histórica multicultural, indica que esses eventos não ocorrem de forma homogênea entre os adolescentes, considerando o sexo, a idade, o tipo de escola em que estudam, a situação, a macrorregião e capital de residência. Os dados mostraram que os comportamentos relacionados ao início da vida sexual e ao uso de métodos contraceptivos na última relação sexual são influenciados pelas características sociodemográficas dos adolescentes.

Mais de um quinto dos adolescentes de 12 a 17 anos de idade já iniciaram a vida sexual no Brasil. A comparação com outros estudos de âmbito nacional é complexa, pois a PeNSE^16^ avaliou apenas escolares do nono ano, isto é, bem mais novos, e a Pesquisa Nacional de Demografia e Saúde de 2006^13^ entrevistou somente mulheres a partir dos 15 anos de idade. Entretanto, pode-se concluir que a prevalência aqui estimada é consistente com esses estudos. A prevalência no Brasil é maior do que a de países de alta renda, como Espanha e Estados Unidos; no entanto, é mais baixa do que em alguns países da África, cuja prevalência alcança 39,0%^5^ ^,^ ^9^ ^,^ ^10^.

A tendência observada de aumento da prevalência de iniciação sexual conforme aumenta a idade também foi confirmada, como observada em outros estudos^4^ ^,^ ^7^ ^,^ ^16^. Aos 17 anos de idade, mais da metade dos adolescentes já havia iniciado a vida sexual, como esperado, uma vez que é um evento que tende a ocorrer a partir dos 15 anos^20^ ^,^ ^21^. É preciso considerar, todavia, que a prevalência de iniciação sexual bem mais baixa observada entre adolescentes de 12 a 14 anos de idade não minimiza sua importância. Pelo contrário, reforça a necessidade de que a educação para a sexualidade deva ocorrer ainda nos primeiros anos da adolescência. Essa ação deve constar nas políticas, programas e práticas na área de saúde do adolescente, no sentido de resguardar seus direitos sexuais e reprodutivos e subsidiar o início da vida sexual saudável, responsável e livre de qualquer coerção.

Tanto a situação de residência (urbana ou rural), quanto a macrorregião de residência, mostraram diferenças nas prevalências de iniciação sexual, sendo os adolescentes da região Norte os que mais relataram ter iniciado a vida sexual. De fato, segundo dados de uma pesquisa nacional^14^, a região Norte apresentou o maior percentual de adolescentes que iniciaram a vida sexual antes dos 15 anos. Resultados da PeNSE 2012 também corroboram a heterogeneidade na prevalência de iniciação sexual segundo a macrorregião de residência^16^. A forma pela qual as características culturais e sociais das diversas regiões brasileiras atuam na tomada de decisão dos adolescentes de iniciar a vida sexual e suas consequências no perfil de saúde desse grupo ainda precisa ser esclarecida.

As prevalências de iniciação sexual de adolescentes foram estatisticamente diferentes para ambos os sexos, o que era esperado, visto ser um evento em que as relações de gênero atuam de forma inquestionável. Os adolescentes iniciam a vida sexual motivados por normas vigentes de comportamentos sexuais, que diferenciam papéis masculinos e femininos no que tange ao momento mais adequado para iniciação sexual^3^ ^,^ ^12^ ^,^ ^20^. A prevalência de iniciação sexual foi maior também entre os estudantes de escolas públicas, o que indiretamente parece ratificar as diferenças entre os estratos socioeconômicos relatadas em outros estudos^7^ ^,^ ^22^.

Quanto à contracepção na última relação sexual, a maioria dos adolescentes referiu ter usado um método contraceptivo, o que também é consonante com outros estudos^1^ ^,^ ^16^. Mensurar o uso de métodos contraceptivos na última relação sexual é uma estratégia eficaz de conhecer o comportamento contraceptivo na adolescência, tendo em vista o caráter esporádico das relações sexuais, a dinâmica própria dos relacionamentos afetivos que se estabelecem nessa fase e a consequente troca e alternância dos métodos. A prevalência de uso de métodos contraceptivos na última relação sexual foi relativamente alta, com diferenças estatisticamente significativas entre sexo, idade e macrorregião, indicando que há grupos sexualmente ativos mais vulneráveis às consequências do sexo desprotegido, como os adolescentes mais novos e os que vivem na região Norte.

No contexto brasileiro, é consenso que o uso de métodos contraceptivos na adolescência limita-se basicamente ao preservativo masculino e à pílula anticoncepcional^3^ ^,^ ^6^ ^,^ ^24^. Nesse sentido, é bastante positivo que a maior parte dos adolescentes tenha usado o preservativo masculino na última relação sexual, como referido em outras pesquisas nacionais;^1^ ^6^ sobretudo por também ser método de prevenção de doenças sexualmente transmissíveis e HIV/aids. Porém, essa prevalência, bem mais baixa que a de 80,0% observada na maior parte dos países europeus^20^, ainda necessita ser ampliada.

Além disso, pouco é conhecido sobre o uso correto e contínuo do preservativo masculino entre adolescentes. Na verdade, no grupo adolescente, observa-se a tendência à flexibilização do uso do preservativo masculino e dos demais métodos quando o relacionamento torna-se estável, ou seja, os adolescentes tendem a ser menos vigilantes quando estão inseridos em relacionamentos mais duradouros^1^. Em concordância, segundo Pirotta e Schor^18^ (2004), o preservativo masculino é usado principalmente nas relações casuais e no início dos relacionamentos sexuais com uma nova parceria, pois os adolescentes podem negligenciar o preservativo masculino em relacionamentos mais duradouros e estáveis, substituindo-o pela pílula hormonal. Se os achados forem analisados na perspectiva da prevenção de gravidez, a alta prevalência de uso do preservativo masculino traduz-se em potencial risco de falha e descontinuidades no uso, visto que a eficácia do método depende da habilidade do indivíduo em usá-lo correta e consistentemente. Isso nem sempre é observado entre adolescentes, principalmente os mais jovens e inexperientes^19^.

Em virtude da preocupação com a prevenção de doenças sexualmente transmissíveis e HIV/aids, os estudos de abrangência nacional mais recentes sobre o uso de métodos contraceptivos entre adolescentes não descrevem o uso das pílulas hormonais, como neste estudo^11^ ^,^ ^16^. Esta é uma potencialidade dos dados sobre comportamento contraceptivo do ERICA frente aos demais estudos nacionais conduzidos com adolescentes. Com exceção do tipo de escola, todas as demais características sociodemográficas foram associadas ao uso da pílula oral no grupo pesquisado.

Mas o achado que mais chama à atenção é a diferença nas prevalências de uso da pílula oral nas regiões Norte e Nordeste em relação às demais macrorregiões. Isso pode ser explicado pelo fato de que um dos elementos fundamentais no uso de métodos contraceptivos é o acesso aos serviços de saúde de qualidade e aos insumos contraceptivos propriamente ditos. O preservativo masculino é de livre acesso na maior parte das unidades de saúde da atenção básica, não requerendo prescrição médica ou qualquer outro pré-requisito para sua obtenção, mesmo por adolescentes. Por sua vez, o processo de obtenção da pílula pode ser mais complexo, pois, como se trata de um método hormonal, sua dispensação no País ainda é permeada pela exigência formal de prescrição médica^15^. No caso específico de adolescentes, essa exigência pode se transformar em barreiras para obtenção do método, a não ser que os serviços estejam suficientemente organizados para contemplar a necessidade por contracepção desse grupo. Nossa hipótese é que, nas regiões onde as barreiras para obtenção da pílula oral não sejam tão evidenciadas, a prevalência de uso da pílula anticonceptiva é maior. Não refutamos, entretanto, que a aquisição da pílula pode ter se dado em farmácias e drogarias comerciais, em que a exigência de prescrição médica é flexível e os preços são relativamente baixos. Nesse caso, fica claro que os adolescentes puderam, de alguma forma, arcar com os custos da compra da pílula, o que nem sempre é possível, a depender do estrato socioeconômico a que pertencem. De toda forma, os achados não nos permitem compreender como foi o processo de obtenção do método contraceptivo nem se houve qualquer tipo de aconselhamento contraceptivo para promover o uso correto e contínuo.

Os resultados revelam singularidades regionais no comportamento sexual e contraceptivo dos adolescentes estudantes brasileiros. A região Norte, por exemplo, apresentou a maior prevalência de iniciação sexual, ao passo que a prevalência de uso de métodos contraceptivos, em especial, da pílula oral, está entre as mais baixas dentre as macrorregiões. As análises por capital, embora nem todas as diferenças tenham sido estatisticamente significativas, também mostram essa preocupante contradição: as capitais da região Norte estão entre as que mais frequentemente os adolescentes reportaram ter iniciado a vida sexual, embora os relatos de uso de métodos contraceptivos, especialmente a pílula oral, não acompanhem a mesma tendência. As políticas de saúde sexual e reprodutiva devem considerar tais diferenças e especificidades regionais na atenção à saúde do adolescente. Programas como o da Saúde na Escola (PSE), que pressupõe a integração entre as escolas e os serviços de saúde, precisam ser inteiramente implementados de forma a contemplar os estudantes e suas necessidades de saúde, pois o ambiente escolar pode ser palco de relevantes transformações na vida e na saúde dos adolescentes. Em última instância, isso poderia contribuir para garantir que as primeiras experiências sexuais dos jovens sejam sexualmente saudáveis, isto é, que sejam desejadas, protegidas e prazerosas.

As limitações deste estudo concentram-se no fato de que certas singularidades do grupo adolescente em relação ao comportamento sexual e contraceptivo envolvem elementos de âmbito individual que não foram explorados, como a idade da primeira relação sexual, o tempo de experiência sexual, o envolvimento afetivo e a estabilidade relacional^1^ ^,^ ^18^. Além disso, embora o ERICA seja um estudo de âmbito nacional, somente os adolescentes estudantes dos municípios de grande porte foram considerados e, portanto, não representa os que residem em municípios com população menor que 100 mil habitantes. Ainda, o ERICA não contemplou os adolescentes que não frequentavam as escolas. Esse fato pode ter acarretado algum tipo de subestimação das estimativas, pois os adolescentes que não estudam podem ser justamente os mais velhos, trabalhadores, ou ainda, mulheres que já vivenciaram a maternidade, isto é, com comportamento sexual distinto.

Adicionalmente, certos comportamentos no âmbito da sexualidade podem ter sido sub ou super-reportados, de forma a atender concepções socialmente aceitáveis. O uso de instrumentos autopreenchidos por meio de PDA pode ter contribuído para amenizar esse viés. De toda forma, trata-se de uma fragilidade inerente aos estudos que contemplam perguntas de cunho íntimo e pessoal entre adolescentes. Não obstante reconhecermos tais limitações, o estudo apresenta importantes contribuições para a temática da sexualidade e contracepção na adolescência.

Concluindo, os dados sobre sexualidade e contracepção do ERICA mostram que há heterogeneidades nas prevalências de iniciação sexual e uso de métodos contraceptivos, como o preservativo masculino e a pílula anticoncepcional oral, entre os adolescentes brasileiros, a depender de sua idade, de onde vivem e do tipo de escola que frequentam. Adolescentes mais novos e residentes na região Norte parecem ser os mais vulneráveis às consequências de práticas sexuais não protegidas.
